# A case report of donor cell–derived hematologic neoplasms 9 years after allogeneic hematopoietic cell transplantation

**DOI:** 10.18632/oncotarget.28686

**Published:** 2025-02-05

**Authors:** Aleksandra Mroczkowska-Bękarciak, Tomasz Wróbel

**Affiliations:** ^1^Department and Clinic of Hematology, Cellular Therapies and Internal Medicine, Wroclaw Medical University, Wroclaw, Poland

**Keywords:** hematology, donor cell-derived hematologic neoplasms, genetics

## Abstract

Background: The treatment of blood cancers has been revolutionized by hematopoietic stem cell transplantation. Owing to this method, we are able to effectively treat most blood cancers. However, in some cases, one of the greatest problems is the risk of relapse. Most often, relapse of the disease manifests itself as cancer cells with the same characteristics as the primary cancer. Nevertheless, a very small percentage of patients develop other blood cancers from donor cells. Donor cell-derived hematologic neoplasms are extremely rare complications that arise after hematopoietic stem cell transplantation.

Case presentation: In this study we described a patient who underwent hematopoietic stem cell transplantation due to acute myeloid leukemia and subsequently developed triple-negative myeloproliferative neoplasms with mutations in the *ASXL1*, *SETBP1* and *EZH2* genes 9 years later. Over the next two years, the disease progressed and MDS/AML developed. Unfortunately, the patient died during induction therapy.

Conclusions: Donor cell–derived hematologic neoplasms are rare but significant complications after HSCT. Early diagnosis and intervention are crucial to improving patient prognosis. Further studies are needed to better understand the pathogenesis of this condition and develop more effective therapeutic strategies.

## INTRODUCTION

An uncommon side effect of allogeneic hematopoietic cell transplantation is donor cell-derived hematologic neoplasm (DCHN), which develops when donor hematopoietic cells undergo oncogenic transformation. Over the past decades, a growing number of cases of donor-derived leukemia have been documented in the literature since the initial report of the condition in 1971 [1]. The true frequency of DCHNs is unknown. The highest prevalence of DCHN was reported in 1982 by Boyd et al. at 1.2% [2]. Later reports have indicated that it was 0.84% by The Tokyo Cord Blood Bank [3]. 0.13% by the Japan Society for Hematopoietic Cell Transplantation [4] and 0.12% by the European Society for Blood and Marrow Transplantation (EBMT) since 2005 [5]. EBMT re-estimated the incidence of donor cell leukemia (DCL) in 2019, and a similar result of 0.1% was obtained [6]. Dietz et al. from the University of Minnesota found that, depending on the graft source, the incidence of DDL ranged from 0.53 to 0.56% at 15 years [7]. The increased use of molecular methods in hematological diagnostics facilitates the precise diagnosis of leukemias originating from donors. Therefore, there have been more reports about this interesting entity recently. The incidence of DCHN increased during a 30-year period from 0.2% to 0.7% according to a retrospective review of 1,994 allogeneic hematopoietic cell transplant recipients [8]. For alloHCT recipients, DCHN has been observed in a number of hematological malignancies, most frequently acute leukemia. DCHN typically has a poor prognosis, with a median survival of a few months to a few years. Therefore, early diagnosis of DCHN is important; moreover, this condition may shed light on host- and donor-derived components involved in the etiology of leukemia [9, 10]. Here, we present a case of a patient who underwent hematopoietic stem cell transplantation (HSCT) due to acute myeloid leukemia (AML) and developed from donor cells triple-negative (TN) myeloproliferative neoplasms with mutations in the *ASXL1, SETBP1* and *EZH2* genes. Over the next two years, the disease progressed, and she developed MDS/AML. A few months after induction therapy, the patient died due to oedema, increasing shortness of breath and infections.

## CASE PRESENTATION

**Figure 1:**
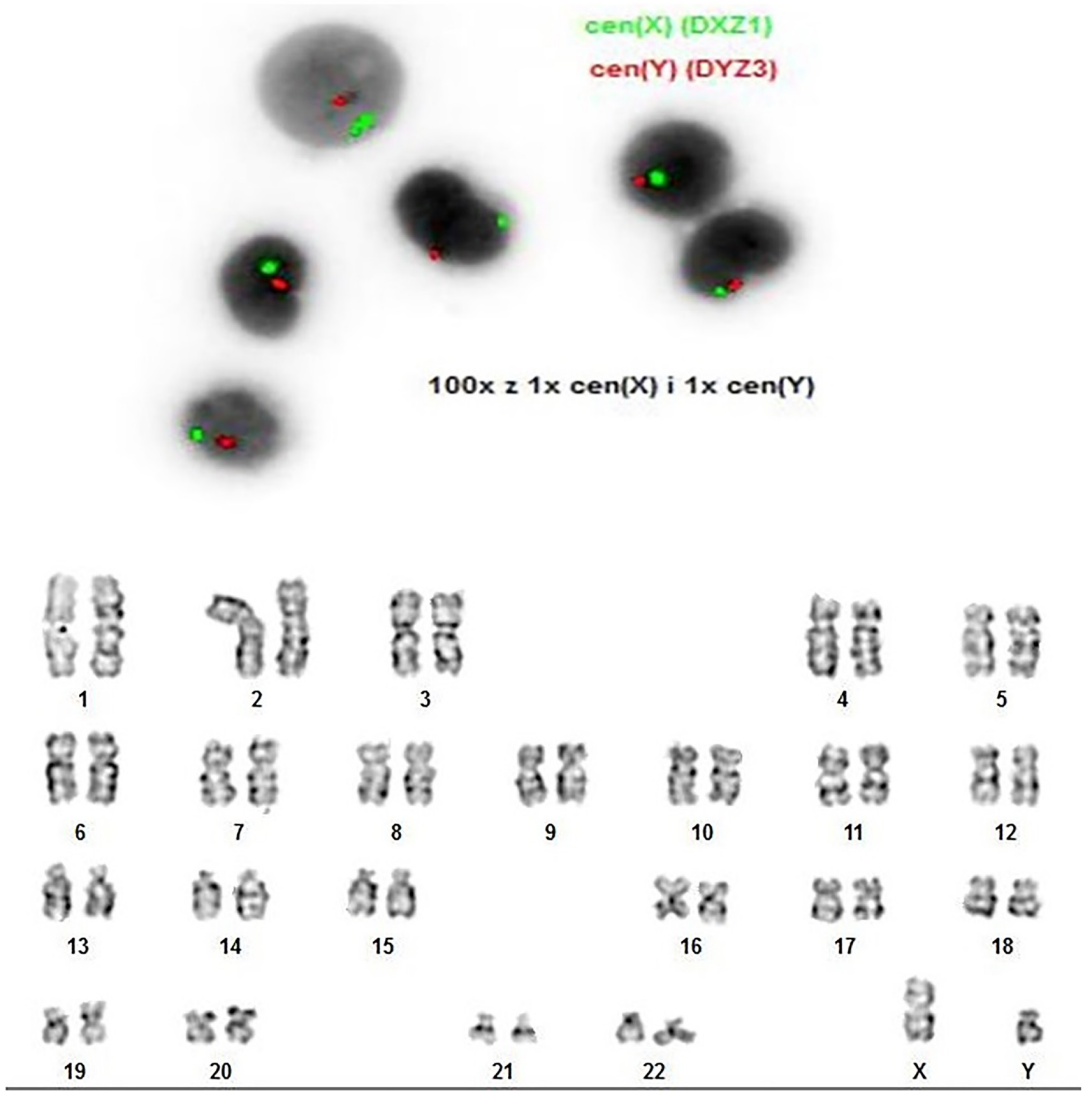
FISH of the sex chromosomes was carried out with the probe sets for the X centromere (CEPX – green) and Y centromere (CEPY – red). Karyotype analysis was performed on a bone marrow sample.

A 23-year-old female with a history of AML who was diagnosed at age 12 with high-risk group (HRG) *FLT3 ITD* positive AML-M4. The presence of a central nervous system (CNS) disease/leukemia was not observed. The patient has been in remission after allogeneic stem cell transplantation from an unrelated donor since 2013. Patient with chronic renal failure, probably due to the toxic effects of CSA or cidofovir, after radical treatment for bladder cancer. She was under the constant care of the Department of Nephrology due to dialysis. In 2022, she was admitted to the hematology department because of an elevated platelet count. To assess posttransplant chimerism, karyotype analysis and fluorescence *in situ* hybridyzation (FISH) were performed. Karyotype analysis was performed on a bone marrow (BM) sample and revealed 46, XY (20). FISH of the sex chromosomes was carried out with probe sets for the X centromere (CEPX) and Y centromere (CEPY), which also showed 100% donor cells (Figure 1). Chimerism test was also performed by QF STR-PCR method using the NGM SElect Human Identification Kit (ThermoFisher Scientific, MA, USA). The study confirmed 100% donor identity. However, to additionally confirm that it was not a relapse of the disease, a PCR fragment analysis test was performed to exclude whether the patient had the *FLT3* variant, as in the original diagnosis. No mutation was detected in the *FLT3* gene. BM examination revealed follicular marrow of medium density with the presence of cellular shadows. The red blood cell system was normal. The granulocytic system was in the upper reference range with a whole series of maturations, and the presence of morphological disorders was mainly in the cytoplasm. The platelet-forming system was characterized by an increased number of megakaryocytes at various stages of development, some with an abnormal nuclear shape. Platelets presented features of anisocytosis and granulation abnormalities. The lymphatic system and the monocyte-macrophage system did not significantly change. Notably, there was an increased percentage of blasts (5.2%) and basophils (3.3%). Additionally, flow cytometric analysis was performed on the diagnostic bone marrow aspirate and showed 5% myeloid blasts. Also, the Ogata score was calculated via four parameters: the granulocyte:lymphocyte SSC ratio, the lymphocyte:myeloblast CD45 mean fluorescence intensity ratio, the percentage of CD34+ B-cell progenitors among the total CD34+ cells and the percentage of CD34-positive myeloblasts among the total nucleated cells. The Ogata score was 4, which indicated dysplasia. Due to the increasing platelet count, molecular tests were performed for suspected myeloproliferative neoplasms. Molecular tests did not detect the presence of mutations in the genes characteristic of myeloproliferative neoplasms such as *JAK2V617F, CALR* and *MPL*. Due to the diagnosis of triple-negative essential thrombocythemia, molecular diagnostics was expanded to include next-generation sequencing to search for a clonal marker of the disease. An AmpliSeq Myeloid Panel was used (MiniSeq, Illumina). The panel detects mutations in 40 genes (23 full genes and 17 hotspot genes), among which mutations occur most often in hematological cancers. Variants were detected via bioinformatic analysis using the BaseSpace Illumina and Variant Interpreter applications in relation to the human hg19 genome. Data analysis identified pathogenic variants in the *ASXL1* and *SETBP1* genes and one pathogenic variant in the *EZH2* gene in the splicing region (Table 1). “Clonal drivers” genes such as *ASXL1* or *EZH2* are one of the most frequently mutated genes in MPNs. These gene mutations occur in all myeloid malignancies and are not specific to MPN. Mutations in the *ASXL1* gene, whose protein product is involved in the regulation of gene expression, have negative prognostic and predictive significance [11]. Missense mutation was identified in the *SETBP1* gene. Mutations in the *SETBP1* gene are associated with an unfavorable prognosis in patients with hematologic malignancies [12]. To exclude an NGS method error caused by technical limitations, the internal tandem duplication mutation in the *FLT3* gene was additionally determined by fragment analysis. The result was negative, which confirmed that it was new leukemia clone. The patient was in good condition and was treated with interferon. She was referred to the hematology clinic, where she was monitored. After less than 2 years, she was readmitted to the hematology department with suspicion of disease progression. A bone marrow biopsy revealed 14% blasts. Molecular tests were performed for the presence of mutations in the *FLT3 ITD, FLT3 TKD* and *NPM1* genes and the presence of the following fusion genes: *BCR::ABL1, CBFB::MYH11,* *RUNX1::RUNX1T1* and *PML::RARA*. The tests performed did not confirm any of the above mentioned genetic changes. The patient was classified as MDS/AML and was treated with venetoclax and azacitidine (VenAZA) at reduced doses due to neutropenia. The patient could not be qualified for a second bone marrow transplant because of kidney failure. Unfortunately, the patient’s condition deteriorated. She required more and more frequent dialysis. Additionally, shortness of breath and oedema occurred. Infections and persistent inflammation (C-reactive protein >300) led to patient death.

**Table 1:** Table showing the results detected by NGS analysis

Gene	Reference sequence	Chromosomal position	cDNA	Protein	VAF (%)	Variant classification
***ASXL1***	NM_015338.5	chr20-31022442--G	c.1934dup	P.Gly646TrpfsTer12	**38,8**	**Pathogenic**
***SETBP1***	NM_015559.2	chr18-42531907-G-A	c.2602G>A	p.Asp868Asn	**39,6**	**Pathogenic**
***EZH2***	NM_004456.4	chr7-148516683-C-G	c.999+5G>C	Splice region	**82,8**	**Pathogenic**

## DISCUSSION AND CONCLUSIONS

The increased use of molecular methods in recent years has accelerated the reporting of DCHN. Because of its ability to shed light on the mechanisms underlying both host- and donor-derived aspects of leukemogenesis, DDMN is a topic of great interest. The only curative or recommended treatment for a number of malignant and nonmalignant hematologic diseases is allogeneic hematopoietic stem cell transplantation (allo-HSCT), the original and most widely used type of cellular therapy. The procedure’s outcome has improved over the past few decades. However, a substantial number of patients experience severe side effects such as graft-versus-host disease (GVHD), infections, or relapse. In fact, relapse of disease, which occurs when diseased cells reappear despite elimination, is the most common cause of death following transplantation [13–15]. Nevertheless, an uncommon but serious consequence is donor cell-derived hematologic neoplasms, which are de novo hematopoietic malignancies formed from donor cells. However, they have acquired genetic alterations that result in a leukemic appearance. These mutations may arise from exposure to mutagenic agents, during the transplantation procedure itself, or during the pre-transplantation stage. Both hereditary and environmental variables play a role in the complicated and many-sided pathogenesis of DCHN. According to the literature, the latency time after donor-derived AML/MDS post-allo-HSCT ranges from 1 month to 24 years, with a median latency of 28 months [9, 15, 16]. Most of the pathophysiology of DCHN mechanisms and risk factors are still unclear. 82 cases of DCHN were found after a study of the literature by Deshmukh et al. The authors analyzed the most cases from donor cell-derived AML and donor-derived MDS. The duration between allo-HCT and DCHN onset varied from 1 to 312 months. A review of the cytogenetic findings showed that monosomy 7 was the most prevalent aberrant karyotype nevertheless a normal karyotype was the most common cytogenetic finding in DCHN. They also reported instances included results that directly addressed the pathophysiology of DCHN. For example the recipient receives a preexisting leukemic clone from the donor, or the donor cell’s innate cytogenetic instability [17]. Another systematic review by Suárez-González et al. shows 137 cases of DCHN. Here the most patients had been diagnosed with AML or ALL . The median time from allo-SCT to DCHN diagnosis was 26 months. They observed similarly to Deshmukh et al. that the most common cytogenetic change was monosomy 7. In contrast, normal karyotype was most frequently reported in donor cell-derived AML /ALL. The authors emphasize that one of the causes of the development of DCHN was that the donor had at least one mutation, which donor passed on to the recipient. However, other genetic or environmental factors, which are typically altered in transplanted patients, have a significant role in the development of malignant disease. Therefore, donors, despite having mutations, do not develop cancer. On the other hand, it may also be the case that preleukemic mutations that do not cause DCHN in the recipient may be present in donors [9]. A malignant clone that was present in the donor has been suggested by several writers as the fundamental cause. In several instances, this theory has been proven true when both the donor and the recipient experienced leukemias with comparable morphologic and immunophenotypic features. Glassier et al. reported a case where acute myeloid leukemia (AML) of donor origin occurred in a 42-year-old woman 18 months after bone marrow transplantation (BMT) from her brother. The donor brother also had AML at the time of DCL, showing the same cytogenetic abnormalities [18]. Zhongwen et al. reported a case of a 28-year-old woman who underwent allo-HSCT for B-ALL. The recipient was diagnosed with *FLT3-ITD*-negative AML 20 months after receiving donor blood stem cells with a pre-existing *ASXL1* mutation. Furthermore, the donor acquired *FLT3-ITD*-positive AML with an identical *ASXL1* mutation 64 months after the recipient’s AML diagnosis [19]. Another theory relies on the donor having a genetic predisposition to hematopoietic malignancies. Familial cancer predisposition syndromes, which are characterized by germline mutations in tumor suppressor or DNA repair genes, genomic instability, and oncogenic mutations, are of particular interest because 80% of donor cell leukemias involve related donors [8, 17]. However, deleterious germline variants in genes such as *CEBPA, DDX41*, or *GATA2* have been associated with both related and unrelated donors. Xiao et al. reported a case of a germline *CEBPA* mutation (584_589dup) in a healthy family donor. In the patient’s microenvironment, susceptible donor hematopoietic cells developed two additional somatic *CEBPA* mutations (247dupC and 914_916dup), leading to overt acute myeloid leukemia [20]. Kobayashi et al. presented case with familial history with hematological malignancies. The patient, a 49-year-old man, was diagnosed with MDS with normal karyotype. He had myeloablative allogenic BM transplantation (BMT) from his HLA-identical sibling. He had thrombocytopenia seventeen months later, and BM aspiration revealed an MDS/EB2 recurrence that progressed to AML. Molecular test showed complete donor chimerism. Both the patient’s and his sibling’s cells underwent whole-exome sequencing. Baccal mucosal swabs were used to isolate and analyze genomic DNA. Analysis reveald a germline mutation in *DDX41* gene in recipient and donor [21]. Galera et al. emphasize that screening for germline mutations in the *GATA2* gene and other genes predisposing to MDS/AML should be performed in all children, adolescents, and young adults diagnosed with MDS/AML, especially if considering the use of a related donor for hematopoietic stem cell transplantation (HSCT). they describe 3 families where each young patient received a transplant from a family donor, then developed MDS/AML again. After several years, the donors also developed hematologic malignancies [22]. Clonal hematopoiesis of indeterminate potential, (CHIP) is the presence of a recurrent somatic mutation in genes linked to myeloid malignancies (*DNMT3A, TET2, ASXL1*) with VAF of >2%. At the same time, the patient has no symptoms of hematological malignancy. The age limit for related donors has naturally increased in tandem with the upper age restriction for hematopoietic stem cell transplantation recipients. The probability of transferring preexisting malignant or premalignant hemopoietic clones during HSCT may increase because aging is a risk factor for cancer [19, 22, 23]. Currently, it is understood that the transplanted cells are subjected to strong demands for proliferation during allo-HSCT, and a high rate of proliferation is frequently associated with an increased risk of replication error or mutation. It is possible for a preleukemic mutation to arise, which could lead to immunosuppression and compromised immune surveillance, thereby increasing the likelihood of hematologic neoplasms [24]. Moreover, damage to niche structures may result from disruptions in the host bone marrow microenvironment following repeated rounds of chemotherapy or bystander radiation, and leukemogenesis may arise from disruptions in the communication between the niche and hematopoietic stem cells [25]. Another study showed that DCHNs are likely caused by damage to the bone marrow microenvironment of allo-HSCT recipients prior to treatment. In one significant series, T-cell depletion seemed to be linked to increased DDM, which could be connected to decreased functional T lymphocytes impairing immune surveillance [6].

Here, we described a patient with a history of acute myeloid leukemia who developed a myeloproliferative neoplasm 9 years after bone marrow transplantation, and in the following two years, the disease progressed, and MDS/AML developed. However, considering the medical history of the patient, who had also undergone treatment for bladder cancer, it can be assumed that the patient’s bone marrow could have been weakened or that the patient’s bone marrow environment created favorable conditions for the development of another hematological cancer. Additionally, the NGS study detected mutations in genes with an unfavorable prognosis, which may predispose to disease transformation. DCHN presents unique clinical consequences and treatment challenges regardless of its rarity. Due to its unpredictable appearance and possible delay, DCHN detection can be difficult and requires careful observation and comprehensive diagnostic testing. After a diagnosis, treating donor cell-derived hematologic neoplasms presents substantial therapeutic challenges that frequently call for immunosuppression, intensive chemotherapy, and, in certa*in situ*ations, a second transplant. The complex nature of DCHN and its influence on patient outcomes require additional research to clarify its etiology, define risk factors, and develop strategies and early detection tactics. Gaining more knowledge about this disease will help with risk assessment, donor selection and the development of innovative treatment strategies tailored to the specific biology of this disease.

## Abbreviations

DCHN: donor cell-derived hematologic neoplasm
DCL: donor cell leukemia
HSCT: hematopoietic stem cell transplantation
AML: acute myeloid leukemia
TN: triple negative
CNS: Central nervous system
FISH: fluorescence *in situ* hybridyzation
BM: bone marrow
GVHD: graft-versus-host disease
